# The Resting‐State Activities of the Angular Gyrus and the Micturition Desire‐Awakening Function in Children With and Without Enuresis

**DOI:** 10.1002/brb3.70177

**Published:** 2024-12-12

**Authors:** Xindi Lin, Shaogen Zhong, Mengxing Wang, Yi Mao, Yuhan Wu, Jiayi Lu, Wei Zhong, Di Wu, Jun Ma

**Affiliations:** ^1^ Department of Developmental and Behavioral Pediatrics, Shanghai Children's Medical Center, School of Medicine Shanghai Jiao Tong University Shanghai China; ^2^ College of Medical Imaging Shanghai University of Medicine and Health Sciences Shanghai China

**Keywords:** angular gyrus, micturition desire‐awakening function, primary nocturnal enuresis, fractional amplitude of low‐frequency fluctuation (fALFF), resting‐state functional connectivity (RSFC)

## Abstract

**Background:**

Micturition desire‐awakening (MDA) function plays a pivotal role in the development of primary nocturnal enuresis (PNE); however, its neural correlates remain largely unexplored. Consequently, this study aimed to identify specific brain regional activities associated with MDA function.

**Methods:**

Neuroimaging data were collected from 173 children with varying MDA functional grade scores at the Department of Developmental and Behavioral Pediatrics, Shanghai Children's Medical Center, from July 2018 to November 2022. Resting‐state images were analyzed using whole‐brain correlation techniques and AlphaSim correction to identify brain regional activities and resting‐state functional connectivity (RSFC) associated with MDA functional grade scores.

**Results:**

Whole‐brain correlation analysis demonstrated that the fractional amplitude of low‐frequency fluctuations in the right angular gyrus (AG) exhibited a negative correlation with MDA functional grade scores (*r*_s_ = −0.336, *p* < 0.001), indicating reduced neural activity in this region with MDA dysfunction. Conversely, RSFC between the right middle frontal gyrus and the right AG was positively correlated with MDA functional grade scores (*r*_s_ = 0.274, *p* < 0.001), suggesting increased connectivity in these areas associated with worse MDA functionality.

**Conclusion:**

These findings provide preliminary insights into the neural underpinnings of MDA functionality.

## Graphical abstract

As illustrated, this study conducted a data‐driven whole‐brain correlation analysis on 173 children at different micturition desire‐awakening (MDA) levels. The analysis revealed that MDA dysfunction was associated with a lower fractional amplitude of low‐frequency fluctuations (fALFF) in the right angular gyrus (AG) (Figure A) and a higher resting‐state functional connectivity (RSFC) between the right AG and the right middle frontal gyrus (MFG) (Figure B).

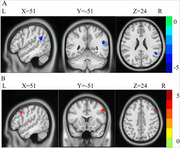

## 1 Introduction

Primary nocturnal enuresis (PNE) is the most prevalent type of lower urinary tract dysfunction, characterized by the inability to maintain urinary control during sleep in children aged 5 years and older who have not achieved a dry period of 6 months or more (Nevéus et al. 2020). This condition results in bedwetting episodes occurring more than once a month and affects approximately 15% to 20% of school‐aged children (Nevéus and Sillén 2013). The pathophysiological mechanisms of PNE are currently believed to include underdeveloped micturition desire‐awakening (MDA) function, reduced nocturnal production of antidiuretic hormone, and decreased bladder capacity (Nevéus et al. 2020; Caldwell et al. 2013).

Among these three mechanisms, an underdeveloped MDA function plays a central role in the pathogenesis of PNE. When nocturnal urine volume surpasses bladder capacity, the occurrence of nocturnal enuresis depends on the maturation of the MDA function. Children with a mature MDA function can wake up and void during the night, thereby preventing enuresis. Therefore, decreased nocturnal production of antidiuretic hormone and limited bladder capacity serve as contributing rather than fundamental factors in PNE. The primary underlying mechanism of PNE is MDA dysfunction (Pedersen et al. 2020; Caldwell et al. 2013).

The achievement of a fully developed MDA function represents a significant developmental milestone in children. Based on extensive clinical observations, we have identified that the development of MDA function typically progresses through five stages: remaining asleep after urination, waking up after completely voiding, waking up after partially voiding a large volume, waking up after partially voiding a small volume, and eventually waking up due to the sensation of needing to urinate. These five stages allow us to accurately assess the developmental status of MDA function (Zhong et al. 2022). However, very few studies to date have investigated the neurobiological mechanisms underlying the development of this crucial function (Zhong et al. 2024). Previous research on the brain structure and function associated with PNE has primarily focused on comparisons between patient groups and healthy controls (HC). For example, children with PNE exhibited reduced thalamocortical resting‐state functional connectivity (RSFC) with the posterior cerebellar lobe, medial and lateral prefrontal lobes, parietal lobe, and precentral gyrus compared to HC (Zhang et al. 2021; Yu et al. 2020; Zhu et al. 2019). Zhu et al. (2019) and Zheng et al. (2021) observed increased regional homogeneity in the left insula, right thalamus, and left superior occipital gyrus, along with decreased amplitude of low‐frequency fluctuations (ALFF) in the left medial orbital superior frontal gyrus and fractional ALFF (fALFF) in the right insula. Additionally, children with PNE showed weakened RSFC between the insula and right superior frontal gyrus (Zhong et al. 2022). A diffusion tensor imaging (DTI) study reported increased mean diffusivity (MD) in the thalamus, frontal lobe, anterior cingulate cortex, and insula, and decreased fractional anisotropy in the thalamus (Lei et al. 2012). Yu et al. (2012) observed lower gray matter (GM) density in the left cerebellum and right dorsolateral prefrontal cortex, while Wang et al. (2018) found increased GM volume in the supplementary motor area and medial prefrontal cortex in PNE children compared to HC. However, these brain abnormalities in PNE may not be directly linked to MDA dysfunction but could instead relate to factors such as bladder capacity, antidiuretic hormone secretion, psychological behavior, comorbidities, and developmental status (Dang and Tang 2021).

To investigate the neurobiological mechanisms of MDA function, we adopted a novel approach that leverages precise clinical assessment techniques to evaluate varying developmental levels of MDA function in children. These stages are then scored and correlated with resting‐state functional Magnetic Resonance Imaging (RS‐fMRI) whole‐brain functional signals (Anticevic et al. 2014). This exploratory analysis aims to identify brain regions associated with MDA function. RS‐fMRI was chosen for its sensitivity and reliability as a probe in brain‐behavior correlation research (Woodward and Cascio 2015). The ALFF measures the intensity of spontaneous regional activities in blood oxygen level‐dependent (BOLD) signals, which indicates robust neural activity in specific brain regions. FALFF was performed to reduce physiological noise and enhance the sensitivity and specificity of ALFF in detecting spontaneous brain activity. First, we conducted whole‐brain correlation analysis between fALFF signals and MDA functional grade scores to identify significant brain regions. Subsequently, we used these regions as seeds for whole‐brain functional connectivity analysis to further explore the correlations between these functional connections and MDA functional grade scores. This study included a substantial sample size with varying levels of MDA functional development. We hypothesized that specific regional brain activities and functional connections between brain areas are associated with the strength of MDA function, prompting this exploratory, data‐driven study.

## 2 Methods

### 2.1 Participants

**FIGURE 1:**
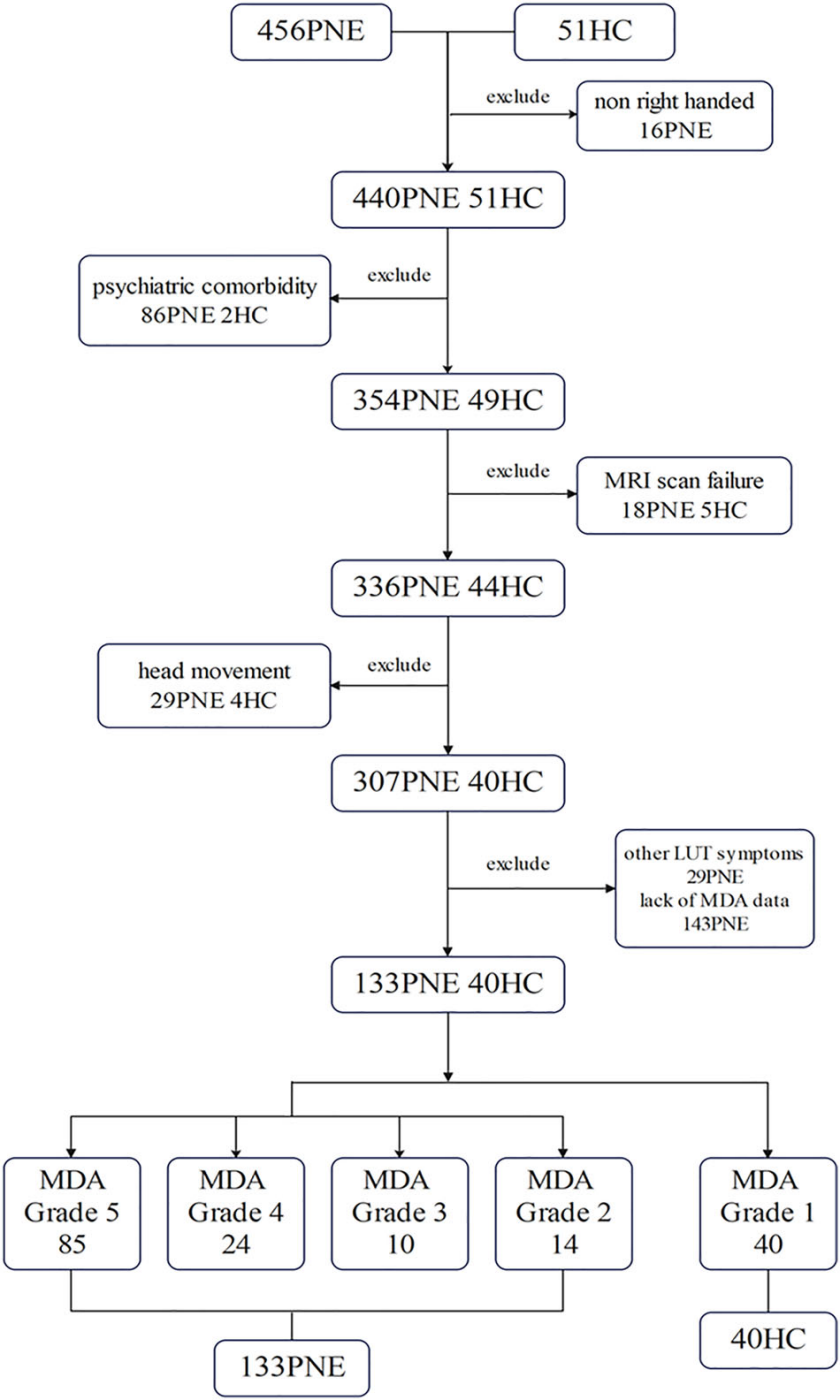
The flowchart depicting participant inclusion and grouping. Abbreviations: HC, healthy controls; LUT, lower urinary tract; MDA, micturition desire‐awakening; MRI, magnetic resonance imaging; PNE, primary nocturnal enuresis.

From July 2018 to November 2022, a total of 456 PNE patients with varying levels of MDA function were randomly enrolled from all children diagnosed with PNE at the Department of Developmental and Behavioral Pediatrics at Shanghai Children's Medical Center. Additionally, 51 age‐ and gender‐matched HC without bedwetting after age 3 and with well‐developed MDA function were first recruited through advertisements and then included via stratified randomization. Based on the Edinburgh Handedness Inventory (Oldfield 1971), all participants reported being right‐handed. Comprehensive clinical evaluations and structured interviews using the Kiddie‐schedule for affective disorders and schizophrenia for school‐age children‐present and lifetime version (K‐SADS‐PL) (Kaufman et al. 1997) excluded any neurological and psychiatric disorders other than PNE. After applying inclusion and exclusion criteria, a total of 173 participants (87 females, age = 8.44 ± 2.14 years) were finally included, as shown in Figure 1. This cohort comprised 133 children with PNE (MDA functional grades: grade 5 [85 children], grade 4 [24 children], grade 3 [10 children], and grade 2 [14 children]) and 40 HC with MDA functional grade 1. The study received ethical approval from the Research Ethics Committee of the Shanghai Children's Medical Center, School of Medicine, Shanghai Jiao Tong University (Approval No. SCMC‐201014), adhering to the principles of the Declaration of Helsinki. Each participant and their parents provided written informed consent prior to participating in the study.

The inclusion criteria for this study were as follows: (1) age range of 5–16 years; (2) right‐handedness; (3) a minimum full‐scale intelligence quotient of 80 on the Wechsler Intelligence Scale for children revised in China (WISC‐RC); (4) HC who met the above criteria and had not been diagnosed with any psychiatric disorders through K‐SADS‐PL interviews; (5) PNE patients diagnosed by at least two developmental‐behavioral pediatricians according to the International Children's Continence Society (ICCS) criteria (Nevéus et al. 2020), with no medical history of conditions related to the onset of enuresis, such as urinary tract infections or diabetes insipidus; and (6) no history of enuresis treatment before MRI scans. The exclusion criteria were: (1) mental or neurological disorders, such as attention deficit hyperactivity disorder (ADHD), tic disorders, intellectual disabilities, or cerebral palsy; (2) MRI scan failures; (3) significant head movement during scans, defined as translation exceeding 3 mm or rotation exceeding 0.2° based on mean framewise displacement (Jenkinson et al. 2002); (4) other lower urinary tract symptoms, such as urinary frequency, urgency, or daytime urinary incontinence; and (5) lack of MDA functional grade scores data.

### 2.2 Clinical Evaluation and Behavioral Assessments

#### 2.2.1 Clinical Evaluation

All participants underwent detailed medical evaluations at the Developmental and Behavioral Pediatrics Department of Shanghai Children's Medical Center. These evaluations included comprehensive clinical interviews (covering chief complaints, current medical history, past medical history, personal history, and family history) and thorough physical examinations (Caldwell et al. 2013). All patients provisionally diagnosed with PNE underwent urinalysis, urinary tract ultrasound, and, if necessary, lumbar‐sacral MRI to exclude potential causes of secondary enuresis, such as urinary tract infections, diabetes insipidus, diabetes mellitus, urethral anomalies, tethered cord syndrome, and occult spinal dysraphism. Additionally, all HC underwent medical examinations to rule out any potential urinary, neurological, or psychiatric disorders. The diagnosis of PNE made by at least two developmental‐behavioral pediatricians according to the ICCS diagnostic criteria for PNE (Nevéus et al. 2020) is as follows: (1) the child must be at least 5 years old; (2) bedwetting occurs at least once a month during sleep; (3) symptoms persist for a minimum of three consecutive months; (4) routine urine tests and urinary system ultrasounds showed no urinary tract infections, diabetes, or other urological abnormalities, nor congenital conditions affecting bladder function; (5) the child has never achieved sustained nighttime dryness (typically longer than 6 months); (6) if necessary, lumbar‐sacral spine MRI scans show no tethered cord syndrome, occult spinal dysraphism, or other organic lesions.

#### 2.2.2 MDA Function Grading System

Based on clinical observations and research on the developmental trajectory of MDA function in children, we have developed a grading system for MDA function. This system aims to accurately capture the stages of MDA function development, particularly in children with PNE, where MDA function is often underdeveloped and at varying stages of maturation (Zhong et al. 2022). MDA function differs significantly from general arousal functions. Previous studies indicate that enuretic children, who fail to awaken due to the need to urinate, do not exhibit impairments in responding to other stimuli, such as visual evoked stimuli (Freitag et al. 2006). In our grading system, MDA function was categorized into five functional grades: Grade 1: waking up due to the sensation of needing to urinate; Grade 2: waking up after partially voiding a small volume; Grade 3: waking up after partially voiding a large volume; Grade 4: waking up after completely voiding; Grade 5: remaining asleep after urination. All participants underwent detailed clinical observations and documentation of MDA functional behaviors by physicians and parents over the course of a month. The majority of exhibited MDA functional behaviors were scored according to this grading system. For HC, parents reported no incidents of enuresis since age three and frequent independent nighttime urination. Over 1 month of clinical observation, these children consistently demonstrated the ability to wake up and urinate after consuming a significant amount of water at night, indicating fully developed MDA function, classified as Grade 1.

#### 2.2.3 Behavioral Assessments

Given that intellectual disability may affect resting‐state brain activity and functional connectivity (Hong 2023), participants underwent IQ testing using the WISC‐RC (Li et al. 1990) with those IQ below 80 excluded. Each participant was interviewed using the K‐SADS‐PL to screen for psychiatric disorders such as ADHD, tic disorders, emotional disorders, schizophrenia, and obsessive‐compulsive disorder (Kaufman et al. 1997). The Edinburgh Handedness Inventory was used to exclude non‐right‐handed children, with younger children completing the inventory with parental assistance. Developmental pediatricians or child psychiatrists with over 10 years of clinical experience conducted the interviews and supervised the questionnaire completion to ensure accuracy in scoring. All behavioral assessors were blinded to the children's enuresis status (single‐blind).

### 2.3 RS‐fMRI Data Acquisition and Preprocessing

#### 2.3.1 Data Acquisition

Structural and functional imaging of the subjects was performed using a 3.0T MRI scanner (Prisma, Siemens, Germany) at the Shanghai Key Laboratory of Magnetic Resonance, East China Normal University, Shanghai, China. During the imaging session, participants were instructed to lie still, relax, close their eyes, and clear their minds (Gusnard and Raichle 2001). Foam pads and earplugs were used to reduce scanner noise and minimize head movement. The fMRI sequence parameters for RS‐fMRI were as follows: volume number = 240, repetition time (TR)/echo time (TE) = 2000/30 ms, acquisition matrix = 64 × 64, acquisition time = 486 s, slice number = 33, flip angle = 90°, voxel size = 3.5 mm × 3.5 mm × 3.5 mm, and field of view (FOV) = 224 mm × 224 mm. The sequence parameters for the high‐resolution T1‐weighted images were acquisition matrix = 256 × 256, inversion time = 1100 ms, TR/TE = 2530/2.98 ms, FOV = 256 mm × 256 mm, flip angle = 7°, voxel size = 1 mm × 1 mm × 1 mm, 192 slices (scan time = 361 s).

#### 2.3.2 Data Preprocessing

All MRI data were preprocessed using MATLAB 2014a (The MathWorks, Inc., Natick, Massachusetts, USA, https://www.mathworks.com/products/matlab, RRID:SCR_001622) through RESTplus version 1.25 (Jia et al. 2019, https://www.restfmri.net, RRID:SCR_009641), a toolkit based on SPM12 (https://github.com/spm, RRID:SCR_007037). Preprocessing steps included: (1) each RS‐fMRI data having its first 10 time points removed to ensure signal stability due to initial scanner calibration; (2) slice‐timing correction to address temporal misalignments caused by the sequential acquisition of image slices; (3) realignment to ensure spatial consistency; (4) normalizing to the Montreal Neurological Institute (MNI) template and resampling into a 3 mm× 3 mm × 3 mm voxel size to ensure consistency in spatial analysis across studies by using T1 image unified segmentation; (5) using a Gaussian kernel (full‐width‐half‐maximum of 6 mm) to smooth to enhance signal‐to‐noise ratio and statistical validity; (6) detrending to eliminate systematic effects over the scanning period; (7) regressing out common covariates, such as cerebrospinal fluid, Friston's 24 head motion parameters, and white matter (WM) signals, to reduce physiological noise effects such as motion fluctuations and respiratory or cardiac fluctuations (Hallquist, Hwang, and Luna 2013); (8) filtering from 0.01 to 0.08 Hz, specifically for RSFC analyses, to focus on the frequency range relevant to neuronal fluctuations but not for fALFF.

#### 2.3.3 Quality Control

Senior developmental‐behavioral pediatricians, imaging experts, and statisticians with extensive research experience collaboratively discussed and formulated the research plan to ensure the study adhered to strict quality standards. A randomized method was used to select a representative sample. Both behavioral and imaging test personnel underwent rigorous training and assessment to ensure high skill levels in conducting the experiment. The data analysis methods were developed with the assistance of experienced statisticians. During the imaging data acquisition phase, foam pads and noise‐canceling headphones were used to improve the success rate of scans. Participants received adequate preparation and practice before resting‐state imaging acquisition, ensuring uniformity across tests. Data not meeting the specified requirements were excluded based on strict criteria to maintain the objectivity and accuracy of the analyzed data.

### 2.4 Statistical Analysis

#### 2.4.1 FALFF‐Behavior Correlation Analysis

Following the method outlined by Zou et al. (2008), the power spectrum at each frequency was calculated and voxel time series were transformed into frequency domain. The root mean square in the low‐frequency range (0.01–0.08 Hz) was computed to obtain the ALFF values. The fALFF values were then derived by dividing the amplitude within the low‐frequency range (0.01–0.08 Hz) by the amplitude across the entire frequency spectrum (0.01–0.25 Hz). To represent the overall neural activity strength of the region, the fALFF was averaged to derive the mean fALFF (mfALFF) values. A whole‐brain correlation analysis was conducted between MDA functional grade scores and mfALFF for each brain voxel, controlling for age and gender. The final results were obtained using AlphaSim correction, with a cluster threshold set at *p* < 0.05 and an individual voxel threshold <0.005, in a brain volume of 61 × 73 × 61, estimated spatial smoothness of 6 mm, and a minimum cluster size of 26 voxels or 702 mm^3^ (AFNI; https://afni.nimh.nih.gov/pub/dist/doc/manual/AlphaSim.pdf). When statistically significant group differences were observed in any brain region, partial Spearman correlation analyses were conducted in R (version 4.2.2, https://www.r‐project.org, RRID:SCR_001905) between mfALFF values and MDA functional grade scores, with age and gender as covariates. A scatter plot of the residuals versus the mean was created to examine the correlation (Pan et al. 2015). Statistical significance was defined as a two‐tailed *p*‐value of less than 0.05.

#### 2.4.2 RSFC‐Behavior Correlation Analysis

Utilizing the specifically identified brain region, the right AG, associated with MDA functional grade scores, RSFC analysis was conducted across the whole brain for each participant. Subsequently, voxel‐based general linear modeling (GLM) was employed to assess the correlation between the seed region and other voxels throughout the brain. The significance of these correlations was determined using AlphaSim correction, with thresholds set at *p* < 0.05 for clusters and *p* < 0.005 for individual voxels, within a brain volume of 61 × 73 × 61, an estimated spatial smoothness of 6 mm, and a minimum cluster size of 26 voxels or 702 mm^3^. RSFC between the right AG and specific brain regions of each participant was transformed into RSFC *z*‐values via Fisher‐*z* transformation. These analyses were performed using RESTplus software. Partial Spearman correlation analyses were then conducted to explore the associations between the RSFC *z*‐values of the right AG and specific brain regions and MDA functional grade scores, with age and sex included as covariates, utilizing R (version 4.2.2, https://www.r‐project.org, RRID:SCR_001905). To assess the correlation between residuals and mean values, a scatter plot was constructed. Statistical significance was established at a two‐tailed *p*‐value of less than 0.05.

## 3 Results

### 3.1 Demographic and Clinical Characteristics

Overall, 173 right‐handed participants with varying MDA functional grades, including 133 PNE patients (8.63 ± 1.75 years) and 40 HC (8.38 ± 2.25 years), were recruited for this study. The MDA grades are presented in Table 1. MDA function was divided into five grades, 40 HC (23.1%) were classified as grade 1, 14 patients (8.1%) as grade 2, 10 patients (5.8%) as grade 3, 24 patients (13.9%) as grade 4, and 85 patients (49.1%) as grade 5. Detailed clinical and demographic data are provided in Table 1. After conducting a chi‐square test, there were no significant differences in gender distribution across the five MDA groups (χ^2^ = 0.696). Analysis of variance (ANOVA) indicated that there were differences in age across the five groups (*p* = 0.027). Therefore, age and gender were controlled as covariates in the relevant analyses.

**TABLE 1:** Differences between MDA functional grades.

MDA functional grade scores	Grade1	Grade2	Grade3	Grade4	Grade5	χ^2^/*p*
Subjects (*n*, %)	40 (23.1%)	14 (8.1%)	10 (5.8%)	24 (13.9%)	85 (49.1%)	NA
Age (mean, SD)	8.63 ± 1.75	7.86 ± 1.56	8.50 ± 3.60	9.63 ± 1.81	8.11 ± 2.66	0.027^a^
Gender (male/female)	23/17	5/9	5/5	11/13	42/43	0.696^b^
AG.R mfALFF (mean, SD)	1.25 ± 0.07	1.26 ± 0.09	1.19 ± 0.12	1.23 ± 0.10	1.17 ± 0.10	<0.0001^a^
AG.R‐MFG.R RSFC (mean, SD)	−0.05 ± 0.24	−0.16 ± 0.18	−0.12 ± 0.25	0.02 ± 0.31	0.02 ± 0.27	<0.0001^a^

Abbreviations: AG.R, right angular gyrus; MDA, micturition desire‐awakening; mfALFF, mean fractional amplitude of low‐frequency fluctuations; MFG.R, right middle frontal gyrus; *n*, number; NA, not applicable; SD, standard deviation; RSFC, resting‐state functional connectivity.

^a^ Participants were divided into five groups based on MDA functional grade scores for ANOVA analysis, denoted here as *p*‐value.

^b^ Participants were divided into five groups based on MDA functional grade scores for a chi‐square test, indicated by *χ* ^2^ value.

### 3.2 Correlation Between FALFF and MDA Functional Grade Scores

**FIGURE 2:**
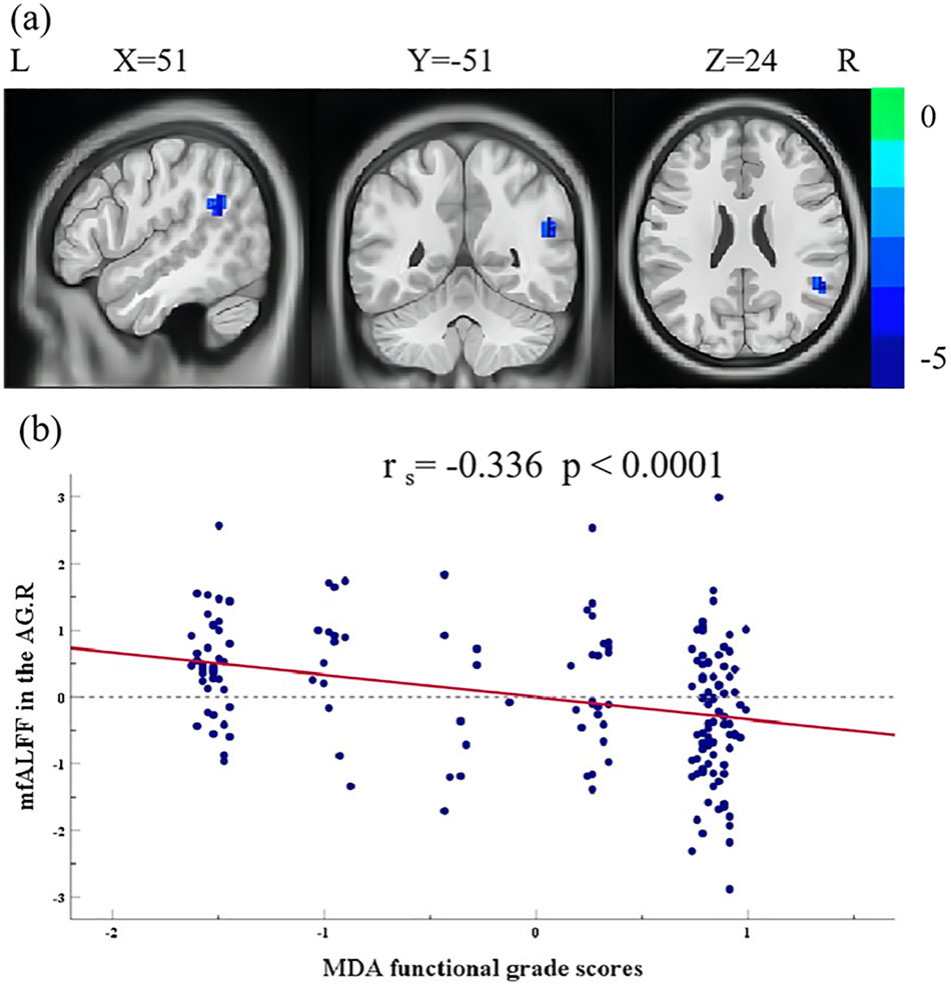
The mfALFF values of brain region associated with MDA functional grade scores after adjusting for age and gender. (a) MDA functional grade scores were negatively associated with the mfALFF in the AG.R. (b) The scatter plot showed the association between MDA functional grade scores and the mfALFF in the AG.R (*r*_s_ = −0.336, *p* < 0.0001). Abbreviations: AG.R, right angular gyrus; MDA, micturition desire‐awakening; mfALFF, mean fractional amplitude of low‐frequency fluctuations.

To reveal the relationship between spontaneous brain activity and MDA functional grade scores, a partial Spearman correlation analysis was conducted between MDA functional grade scores and the mfALFF of each voxel in the brain. After adjusting for gender and age, MDA functional grade scores were negatively related to the mfALFF in the right AG (Figure 2 and Table 2) (*r*_s_ = −0.336, *p* < 0.0001).

**TABLE 2:** Brain regions showing significant association with MDA functional grade scores.

Region	BA	Cluster size (mm^3^)	MNI coordinates			Peak *Z* value
			*X*	*Y*	*Z*	
mfALFF						
AG.R	40	810	51	−51	24	−3.9344
RSFC *z*‐values						
MFG.R	NA	918	51	15	42	3.8429

*Note*: After applying partial Spearman correlation and AlphaSim correction, the brain region associated with mfALFF and MDA functional grade scores was identified as the AG.R, and the brain region associated with RSFC *z*‐values and MDA functional grade scores was identified as the MFG.R. AlphaSim correction was applied with *p* < 0.005 at the voxel level, minimum cluster size of 702 mm^3^, corresponding to a significant corrected threshold of *p* < 0.05.

Abbreviations: AG.R, right angular gyrus; BA, Brodmann's area; FC, functional connectivity; MDA, micturition desire‐awakening; mfALFF, mean fractional amplitude of low‐frequency fluctuations; MFG.R, right middle frontal gyrus; MNI, Montreal Neurological Institute; NA, not applicable.

### 3.3 Correlation Between RSFC and MDA Functional Grade Scores

**FIGURE 3:**
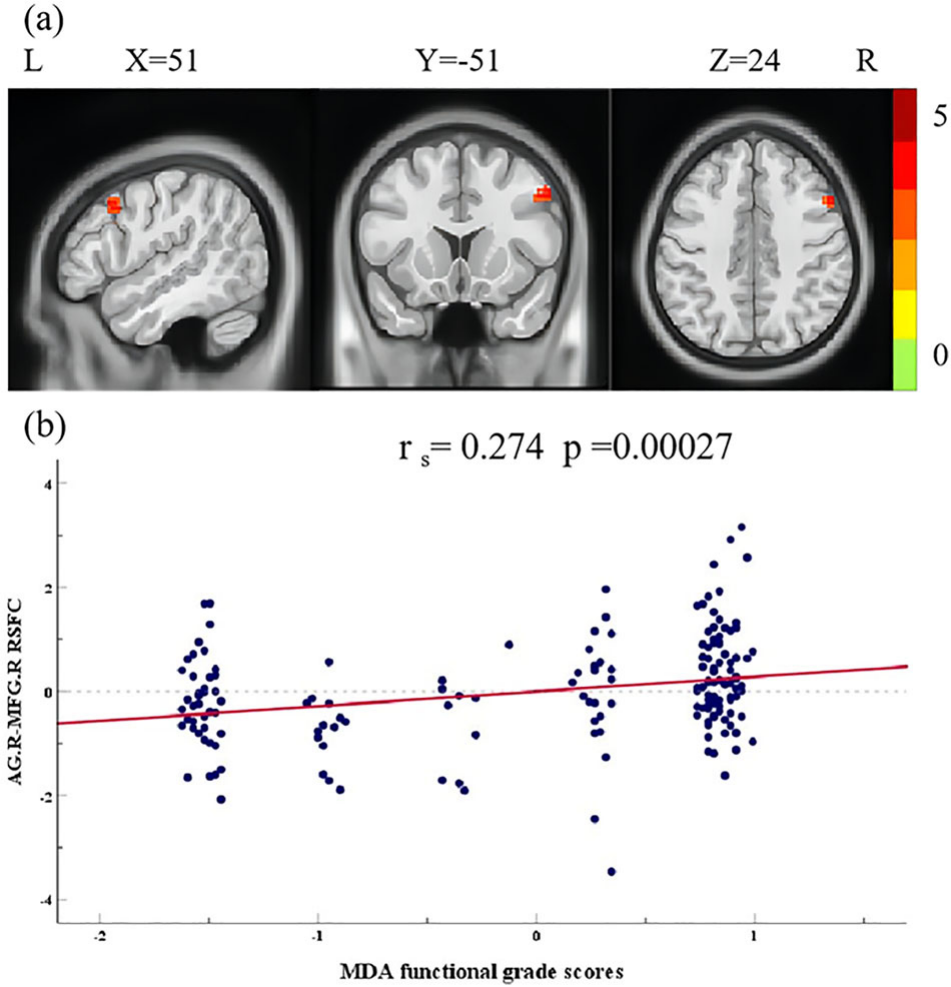
The RSFC values between AG.R and MFG.R associated with MDA functional grade scores after adjusting for age and gender. (a) MDA functional grade scores were positively associated with the RSFC *z*‐values between AG.R and MFG.R. (b) The scatter plot showed the association between MDA functional grade scores and the RSFC *z*‐values of AG.R‐MFG.R (*r*_s_ = 0.274, *p* = 0.00027). Abbreviations: AG.R, right angular gyrus; MDA, micturition desire‐awakening; MFG.R, right middle frontal gyrus; RSFC, resting‐state functional connectivity.

To explore whether interactions between the right AG and other regions were correlated with MDA functional grade scores, a partial Spearman correlation analysis was conducted between MDA functional grade scores and RSFC *z*‐values, controlling for gender and age. The analysis revealed a positive correlation between MDA functional grade scores and the RSFC *z*‐values between the right AG and the right MFG (*r*_s_ = 0.274, *p* = 0.00027), as demonstrated in Figure 3 and Table 2).

## 4 Discussion

This study is an exploratory whole‐brain correlation study. Through this data‐driven approach, we found that only lower fALFF in the right AG was associated with MDA dysfunction, while higher RSFC between the right AG and the right MFG was also associated with MDA dysfunction. These results support our hypothesis that functional activity and connectivity in specific brain regions are related to different developmental levels of MDA function, suggesting that the right AG and its RSFC with the right MFG play important roles in the development of MDA function.

The AG is essential for a variety of functions, including transitions between sleep and wakefulness, information processing, attention, spatial cognition, language, and working memory (Seghier 2013). Moreover, the AG serves as a crucial hub for cross‐modal integration within several key brain networks, including the DMN, the frontoparietal network, and the dorsal attention network (DAN). The activity of the DMN persists during light sleep and gradually decreases with the deepening of sleep, significantly diminishing at the deepest stages of sleep, when arousal thresholds are highest (Horovitz et al. 2009). As sleep deepens, both AG and DMN activity decrease, potentially affecting MDA function and making it harder for children to awaken in response to the sensation of needing to urinate. This phenomenon may partly explain the association observed in our study between reduced fALFF in the right AG and MDA dysfunction. Studies by Griffiths (2015) and Pang, Gao, and Liao (2022) indicated that the DMN is activated during urination control in healthy individuals, suggesting that children with diminished local activity in the AG and reduced DMN activity may have difficulty controlling urination at night.

This study also found that increased RSFC between the right AG and right MFG is associated with worsening MDA functionality. The MFG, located in the frontal lobe and nestled between the superior and inferior frontal sulci, is a junction of both the ventral attentional network and the DAN. Current research has confirmed that the MFG plays a significant role in emotional control, sensory processing, and cognitive functions (Seminowicz and Moayedi 2017). Additionally, related studies indicate that the MFG is involved in urinary control. For instance, Mehnert et al. (2008) discovered that stimulation of the dorsal clitoral nerve during bladder filling reduced bilateral MFG activation, thereby enhancing bladder control. Consequently, increased MFG activity may lead to weakened bladder control and premature urine discharge. MDA functionality can essentially be divided into three components: the transmission of bladder fullness and the sensation of the need to urinate to the brain, the brain's processing of these signals to generate awareness, and the control of bladder emptying until the individual awakens and urinates. As a result, increased RSFC between the MFG and AG may weaken bladder control, leading to premature urinary discharge (Nour et al. 2000; Zuo et al. 2019) and reduced MDA functionality. Consequently, enhanced RSFC between the MFG and AG may represent an abnormal neural circuit contributing to MDA dysfunction. Alternatively, another possible explanation is that when AG activity decreases, leading to reduced MDA functionality, the MFG may attempt to compensate by enhancing RSFC with the AG to restore MDA function. However, this compensatory pathway may be ineffective, and as MDA function weakens, RSFC between the MFG and AG becomes more pronounced. This relationship may explain the observed association between right MFG‐right AG RSFC and MDA dysfunction in this study. Nevertheless, this mechanism cannot be confirmed by the present study and requires further in‐depth investigation, such as animal studies, to elucidate the underlying mechanisms.

To date, no PNE studies have directly linked the AG and MFG to the core mechanism of enuresis: MDA dysfunction. Our research expands the preliminary understanding of the neural mechanisms underlying MDA dysfunction, providing a foundation for further studies to identify the neural networks involved in MDA functionality in the human brain. This study has several advantages: (1) It includes a substantial sample size, allowing precise behavioral scoring based on various developmental levels of MDA function, which helps capture subtle MDA behavioral differences and identify robust correlates of brain function. (2) We conducted whole‐brain correlation analyses of MDA functional grade scores, identifying the brain characteristics most closely related to MDA function by examining signals from all voxels, ensuring no significant regions were overlooked. (3) We discovered a novel correlation between the right AG and MDA function, enhancing understanding of the right AG role not only in sleep‐wake regulation but also in controlling urination. However, there are several limitations: (1) Although substantial, the sample size may still be insufficient to detect smaller brain regions related to MDA function, suggesting a need for larger future studies. (2) The cross‐sectional design prevents inference of causality between brain activity, functional connectivity, and MDA function, necessitating prospective cohort studies and animal experiments to clarify these relationships and elucidate the mechanisms of MDA function development. (3) Despite the rigorous design and clinically representative samples, the results may not generalize to children of diverse ethnicities and countries, highlighting the need for studies investigating MDA function across populations. (4) The potential role of the right AG and right MFG as either an abnormal neural circuit or a compensatory pathway remains speculative and requires further basic research for validation.

## 5 Conclusion

In conclusion, this study found that regional activity in the right AG and its RSFC with the right MFG were correlated with the development of the MDA function at various stages. These findings offer valuable insights for future research into the neural mechanisms underlying MDA function.

## Author Contributions

**Xindi Lin**: conceptualization, writing–original draft, data curation, formal analysis, methodology. **Shaogen Zhong**: conceptualization, methodology, data curation, formal analysis, validation. **Mengxing Wang**: data curation, investigation, supervision. **Yi Mao**: validation, supervision. **Yuhan Wu**: supervision, validation. **Jiayi Lu**: validation, supervision. **Wei Zhong**: validation, supervision. **Di Wu**: validation, supervision. **Jun Ma**: conceptualization, supervision, funding acquisition, project administration, resources, writing–review and editing, writing–original draft.

## Conflicts of Interest

The authors declare no conflicts of interest.

### Peer Review

The peer review history for this article is available at https://publons.com/publon/10.1002/brb3.70177.

## Data Availability

The data that support the findings of this study are available on request from the corresponding author. The data are not publicly available due to privacy or ethical restrictions.
